# Ten simple rules to aid in achieving a vision

**DOI:** 10.1371/journal.pcbi.1007395

**Published:** 2019-10-17

**Authors:** Philip E. Bourne

**Affiliations:** 1 School of Data Science, University of Virginia, Charlottesville, Virginia, United States of America; 2 Department of Biomedical Engineering, University of Virginia, Charlottesville, Virginia, United States of America; Dassault Systemes BIOVIA, UNITED STATES

*Whoever has visions should go to the doctor*.

Helmut Schmidt [1]

In a career that now spans 40 years, I have had, on several occasions, opportunities to turn loose ideas into a unified vision and the resources to implement that vision. What do I mean by a vision? A vision, at least in my mind, is the ability to see something important to the future, perhaps before others do. Fulfilling that vision does not have to change the whole world (although that would be nice) but only to impact others in a positive way. Consider what I perceive has been my own visioning to provide some context.

My visioning began in the 1990s when, seeing what computation was bringing to the life sciences through work on the human genome project, I was lucky enough to be able to envision and establish a bioinformatics laboratory before the idea became mainstream. In the early 2000s, it was a collective vision for what an exemplar data resource, namely, the Research Collaboratory for Structural Bioinformatics (RCSB) Protein Data Bank (PDB), should achieve. Around 2005, it was something dear to this readership, a vision for a new journal, *PLOS Computational Biology*, for which I was cofounder and Founding Editor-in-Chief for 7 years. Around 2007, it was forming a company, SciVee.tv, to envision how digital media other than print could be used to communicate science. Around 2014, as the first Associate Director for Data Science (ADDS) for the United States National Institutes of Health (NIH), the vision was how big data could catalyze change in life sciences research. Finally, now in 2019, the vision is how one of the first academic Schools of Data Science should be established and run. These either were, or are, great opportunities to lay out a vision and act upon that blueprint. Not all were successful (*PLOS Computational Biology* was), but all were learnt from. So, what did I learn? Here is at least part of my life’s lesson, in that now familiar and comfortable Ten Simple Rules format. In this article, the rules are generic and can be considered beyond our own discipline.

## Rule 1: Be realistic

I went to the US NIH with a vision to create a truly interdisciplinary environment, breaking down the silos (i.e., cylinders of excellence) that exist in individual institutes using data sharing as a catalyst. This was an unrealistic vision, given that such a change was not about better technologies; it was about a change in culture, and cultural changes typically occur slowly and in increments. Fulfilling a vision requires being realistic as to what can be accomplished and in what timeframe, yet it also means seeing beyond the current culture. It is all too easy to accept the status quo, and doing so will not allow you to envision anything but simply be incremental in your thinking. So, it is necessary, in the case of the NIH and indeed any academic institution, to first continually and consistently communicate your vision (see Rule 6) and figure out ways to slowly introduce components of the overall vision that are easily seen to make sense to the organization writ large.

## Rule 2: Truly believe in your vision

If you don’t wake up thinking of how to fulfill your vision, you are likely not going to. That waking thought is a reflection of your passion and belief. The passion you display will be associated with many of the other rules that follow. If what you propose truly is visionary, there will be many who will be the first to tell you why it is a bad idea. If your belief falters based on their input, you are not going to achieve the vision. Your own convictions begat the strength and courage to move forward against the odds. To quote one example from my own experience, when we took over the RCSB PDB from the Brookhaven National Laboratory, there were naysayers both verbally and in print who said this was “the end of structural biology.” There was great scrutiny and community pressure to succeed. If we had not truly believed in our collective vision (see Rule 7), we could have faltered.

## Rule 3: Have a plan to fulfill the vision and expect both to change

A vision must be more than a vague idea. It will start that way, but if you can’t very quickly write down a plan for how to fulfill that vision, it likely is not going to amount to much. You certainly won’t have it all worked out at the outset, but if you can’t build on the basic concept as you begin to popularize and evangelize your vision (see Rule 6), then it is likely doomed. As you do start to flesh out the vision, the passion (as defined by Rule 2) should increase. If it diminishes, you are in trouble. The vision will also morph as others weigh in and get involved. That is usually a good thing as long as that influence enables you to see the vision more and not less clearly. Another outcome is how time morphs your vision. Case in point was this journal. The original vision was to get experimentalists to read and adopt the work of computational biologists. I am not convinced that happened to the degree hoped; what did happen was that, over time, we saw an ever-increasing number of scientists trained in computation and experiment. The end result was the same: success—a highly sought-after journal, with lots of other innovations that were not part of the original vision (this Ten Simple Rules series being an unforeseen example).

## Rule 4: Be in the right place at the right time and seize the opportunity

I have pitched a vision for a company in the hope of getting funding on the very same day that another company doing much the same publicly announced, with a big splash, their first round of funding. We were much too late to the marketplace. Conversely, being too early will likely not enable others to see the vision—there is just not enough context for others to grasp, regardless of how hard you work on Rule 6. However, don’t forget those “too early” visions completely; their time may come. I wish I had advice for knowing what is the right place and time, but I do not. It’s mostly luck. In a crude way, it relates to Rule 5. If you can’t fund the vision, then it is not the right place or time.

## Rule 5: Be able to finance your vision

A vision will likely never be realized without money. Without money, it will be a hobby at best. This is certainly true of a scientific vision. Money can come from grants, contracts, angel or venture capital investment, philanthropy, and I am sure other sources I have yet to imagine. A vision is a big idea, and it will cost. Rule 6 comes into play here. Others have to see the value in your vision, whether they be grant reviewers, big time venture capitalists, philanthropists, or others. The vision needs to align with the requirements of the funding source. Anyone who has written and then not gotten a grant knows this. Less obvious is alignment with other funding sources. For example, when we tried to raise money from venture capitalists with what we thought was a great vision for a new form of scientific communication, they did not agree, asking “where is the hockey stick?” implying they did not see the financial growth potential after an initial investment. We showed only a steady growth, not the next Google or Facebook. On the other hand, we just received the largest gift in our university’s history as our vision for a School of Data Science was in alignment with that of the donor foundation. That alignment often comes from trust built over time.

## Rule 6: Be able to communicate the vision effectively

Chances are, a meaningful vision will be outside of the scope of what those who can carry it forward can easily comprehend, otherwise they would have thought of and possibly implemented that vision themselves. A clear articulation of what your vision is, how it will be implemented, and most importantly, what positive effect it will have are critical. Again, drawing on my own experience, our articulation of the NIH Data Commons [2], which abided by the Findable, Accessible, Interoperable, and Reusable (FAIR) data principles [3], illustrates this rule. It also illustrated the importance of communication to all stakeholders—from institutional leadership in supporting the vision to those who are the beneficiaries of its implementation. As an aside, the vision for the NIH Data Commons also brings up two other points, the first related to Rule 1. The NIH Data Commons was only part of what we envisioned for the NIH and came from being realistic. Second, the vision does not necessarily need to be a completely new idea—the Genomic Data Commons [4], for example, already existed.

Articulation of the vision comes in multiple forms—written and verbal and in multiple media, formal peer-reviewed articles, podcasts, social media, slides and associated presentations, and video. Once you have articulated the vision, keep doing so. If the vision makes sense, sooner or later, others will start to articulate the same vision as if it were their own or, indeed, articulate an even better vision, which takes us to Rule 7.

## Rule 7: Be willing to share ownership

Although you might have the original vision, you will not carry it out alone. Sharing the vision also involves being willing to share the credit for that vision or, indeed, to gain no credit for that vision at all. If you are not willing accept these outcomes, chances are your vision will die on the vine. Sharing with others who have their own visioning skills will amplify the value of what you foresee. Once shared, suitable partners will also communicate the vision, and so the value of the vision compounds while your contribution as an individual diminishes. Sharing is antithetical to the academic system that is geared (alas) towards rewarding the individual, and so traditional reward systems may not apply. For the visions I have been involved in, they just would not have happened without sharing and collaboration. Consider my latest attempt at a vision: envisioning a new type of school in a conservative academic setting. If successful, this will be the result of senior leaders, donors, and a team that shares the original vision, shares their additive contributions to the vision, and pulls together as a team.

## Rule 8: Be patient and don’t expect rewards

The greatest of visions takes time to be adopted. A vision is typically not a single discovery, although such a discovery might be foundational to that vision. The structure of DNA was foundational, the discovery of CRISPR-Cas9 was foundational, but the vision lies in how those discoveries can be broadly used, which takes time to be appreciated (in these examples, genomics more broadly and the possibilities of gene editing, respectively). Science typically recognizes discoveries but not necessarily the vision to utilize those discoveries. The reason, as already stated, is that science typically rewards individuals but not a collective vision. Stated another way, discovery belongs to a few individuals; visions that build upon those discoveries are many and belong to many people.

## Rule 9: Know when to stop

You may have a great vision, but it becomes clear that it is just not going to happen. The reasons can be many. One I have experienced a number of times and raised here in Rule 1 and elsewhere relates to a vision that requires a change in culture. Changing culture in anything less than a generation is hard. It can happen, but the incentives have to be there, or you have to be able to find ways to change the incentives, which also can be very hard. Consider interdisciplinary research, which cuts across a number of my own visions. Most would agree that there is much to be gained from interdisciplinary research, but still, outdated models of funding and university organization hamper interdisciplinary activity. Money still flows to the traditional silos; any vision that seeks to break that model and the culture it implies will be hard pressed. That does not imply that one should give up on the vision; just be more realistic as to the time it will take. Knowing which visions are a lost cause as they will never happen and those that will take a long time is something I am still learning. Looking back, I would say that when the pace of seeing your vision come to fruition is so slow as to have the frustration it causes impact the rest of your life, it is time to stop and take up a new vision.

## Rule 10: Take time to enjoy the experience

Fulfilling a vision requires immersion in the process of making that vision a reality. This can become all-encompassing to the point that when one milestone is reached, rather than taking the time to relish that success one rushes on to the next challenge. It’s easy to become obsessive and not enjoy the creative experience. In accordance with Rule 7, you will have others with whom to share the experience. Take the time to do so. It builds the team and makes it all worthwhile. In our latest vision for establishing a School of Data Science, we celebrate each milestone together with those who have made it happen. This takes the form of dinners, sometime at my house, which speaks to a commitment of time and the notion of family. In the case of *PLOS Computational Biology*, it is the annual dinner for editors as an annual meeting. In the case of the lab, it is hikes, fishing, skiing, and other social occasions to celebrate together and with our families.

We all envision; we would not be scientists if we did not. Visions come at different scales and in different forms. Certainly, these rules are neither sufficient nor will they cause you to fulfill your visions. Hopefully, they will at least get you thinking about the visioning process, which is different for each one of us. To that end, the rules are generic and broadly applicable across science and, indeed, society.

As a scientist, all I can do is offer my own experiences and suggest that, although the body of work in your next paper is a vision of sorts, you should look beyond that to a body of work, the translation of that body of research into product, and the influencing of your whole field. As a society, our future depends on it.
